# Antimicrobial and Antioxidant Activities of Essential Oils of *Satureja thymbra* Growing Wild in Libya

**DOI:** 10.3390/molecules17054836

**Published:** 2012-04-26

**Authors:** Abdulhmid Giweli, Ana M. Džamić, Marina Soković, Mihailo S. Ristić, Petar D. Marin

**Affiliations:** 1 Institute of Botany and Botanical Garden “Jevremovac”, Faculty of Biology, University of Belgrade, Studentski trg 16, 11000 Belgrade, Serbia; Email: simicana@bio.bg.ac.rs (A.M.D.); 2 Department of botany, Faculty of Science, University of Al-Gabel Al-Garbe, Zintan, Libya; Email: zen_giweli@yahoo.com; 3 Mycological Laboratory, Department of Plant Physiology, University of Belgrade-Institute for Biological Research “Siniša Stanković” Bulevar Despota Stefana 142, 11000 Belgrade, Serbia; Email: mris@ibiss.bg.ac.rs; 4 Institute for Medicinal Plant Research “Dr Josif Pančić”, Tadeuša Košćuška 1, 11000 Belgrade, Serbia; Email: mristic@mocbilja.rs

**Keywords:** *Satureja thymbra*, essential oil, antimicrobial, antioxidant activity, γ-terpinene, thymol, carvacrol

## Abstract

The composition of essential oil isolated from *Satureja thymbra,* growing wild in Libya, was analyzed by GC and GC-MS. The essential oil was characterized by γ-terpinene (39.23%), thymol (25.16%), p-cymene (7.17%) and carvacrol (4.18%) as the major constituents. Antioxidant activity was analyzed using the 2,2-diphenyl-1-picrylhydrazyl (DPPH) free radical scavenging method. It possessed strong antioxidant activity (IC50 = 0.0967 mg/mL). The essential oil was also screened for its antimicrobial activity against eight bacterial and eight fungal species, showing excellent antimicrobial activity against the microorganisms used, in particular against the fungi. The oil of *S. thymbra* showed bacteriostatic activity at 0.001–0.1 mg/mL and was bactericidal at 0.002–0.2 mg/mL; fungistatic effects at 0.001–0.025 mg/mL and fungicidal effects at 0.001–0.1 mg/mL. The main constituents thymol, carvacrol and γ-terpinene also showed strong antimicrobial activity. The commercial fungicide bifonazole showed much lower antifungal activity than the tested oil.

## 1. Introduction

Species of the genus *Satureja* (family Lamiaceae) are widely distributed in the Mediterranean area, Asia and boreal America, regularly found in sunny, dry, rocky habitats. *Satureja* consists of about 200 species, usually aromatic herbs and shrubs. In Libya, this genus is represented by only two species—*S. thymbra* and *S. fortii* the second being endemic to Libya. The two species could be found only in the Green Mountains (Eastern Libya) [1]. *S. thymbra* L. is a very branched, usually grey-puberulent dwarf shrub, 20–35 cm [2]. In Europe, this species is distributed in the South Aegean region and the south coast of Sardinia.

A number of studies have focused on the chemical characteristics of natural flora of a specific region or country. This criterion may be useful for understanding the activity of plants used in folk medicine in different parts of the World, and it is a valuable as an ethnopharmacological criterion, which is important in characterization of some oil chemotypes [3]. The essential oil of this species is used in folk medicine as an antiseptic, tonic, gastric sedative and diuretic [4]. Also, the aerial parts of some *Satureja* plants have been widely used in folk and traditional medicine, to treat many ailments, for instance muscle pains, indigestion, cramps, nausea, diarrhea and infectious diseases [5,6]. In addition, they are used traditionally as an antibacterial for the treatment of cold and bronchitis [7].

*S. thymbra* oil has been found to have a good antimicrobial activity against various bacteria and fungi [7,8,9]. It has also been evaluated for inhibitory activity against SARS-CoV and HSV-1 replication *in vitro* by visually scoring of the virus-induced cytopathogenic effects post-infection [10]. The essential oil of *S. thymbra* (1%), as well as its hydrosol fraction (100%). have also been reported to present strong bactericidal effects against bacterial biofilms formed on stainless steel by some useful, technological and pathogenic bacteria [11].

Recently, the acaricidal activity of *S. thymbra* essential oil and its major components carvacrol and γ-terpinene were tested against adult *Hyalomma marginatum* (Acari: Ixodidae) [12]. In addition, insecticidal activity of the essential oils from this and related plant species was evaluated against three stored-product insects [13]. 

It has been shown that essential oil content and composition is related to genetic [14], edaphic, climate, topography, altitude [15,16], genotype and growing conditions [17]). Different chemotypes have been described for a number of medicinal plants [14,18,19,20]. Also, several recent results have shown that some medicinal plant characteristics can be affected by ecological factors such as temperature, plant competition, nitrogen content in the soil and precipitation [21]. 

So far, several articles were published dealing with the essential oil of *S. thymbra* [8,10,22,23,24,25,26,27,28,29]. Its constituents were determined to be mainly carvacrol, thymol, *p*-cymene, and γ-terpinene. However, significant differences were reported in essential oil composition in samples from different regions. In addition, according to recent results of essential oil composition of this species collected from two locations in Greece, the authors found different chemotypes [24].

These aspects of many medicinal plants from Libyan flora are still insufficiently investigated. To the best of our knowledge, there are no previous reports on chemical composition and biological activity of essential oil of *S. thymbra* originating from this area. The aims of this study were to characterize the composition of the essential oil of wild-growing *S. thymbra* from Libya and compare it with other reported results of samples of this species collected from various areas of its natural distribution, since different oil compositions were published so far. The goal was also to test the antioxidant, antifungal and antibacterial activities of the analyzed essential oil as a potentially new source of biologically active natural products. 

## 2. Results and Discussion

The composition of the essential oil isolated from *S. thymbra* is given in Table 1. The essential oil was characterized by γ-terpinene (39.26%), thymol (25.16%), *p*-cymene (7.17%) and carvacrol (4.18%) as the major constituents. Our findings are in contrast with some previous observations of *S. thymbra* essential oil [10,13,22,23,24,25,26,27,28,30,31]. In most references mentioned, carvacrol and/or thymol were the major compounds. Thus, the essential oil composition of *S. thymbra* from Turkey contained mainly carvacrol and γ-terpinene [25]. *S. thymbra* essential oil from Lebanon was characterized by similar amounts of *p*-cymene (10.76%), α-pinene (10.15%), thymol (9.92%) and sabinene (8.64%) [10]. According to the essential oil composition of this species collected from Crete (Greece) from two locations, it presented different chemotypes. In the sample from Aktotiri the major compounds were carvacrol, thymol, *p*-cymene and γ-terpinene (44.6%, 0.3%, 11.9% and 25.5% respectively), while in the other sample the main compounds were thymol (35.5%), γ-terpinene (27.6%), *p*-cymene (10.4%) and carvacrol (3.2%) [24]. The essential oil of the same species growing wild in Turkey was also dominated by carvacrol (53.7%), followed by γ-terpinene (17.6%), thymol (13%) and *p*-cymene (10.1%) [13]. Furthermore, in a study of the essential oil composition of samples from 13 essential oils of *S. thymbra* from different locations in Crete, the findings showed a high compositional variation in which carvacrol varied significantly (5.2–65%), thymol (0.1–65.6%), γ-terpinene (20–4.4%) and cymene (5.5–15%) [22]. Moreover, a study was carried out to evaluate the effect of harvesting time on essential oil composition of *S. thymbra* collected from the same location at different vegetation stages. The findings showed that the major compounds in the vegetation phase were thymol (27.88%), followed by γ-terpinene (17.02%) and carvacrol (11.88%), while in full flowering phase carvacrol (29.18%), thymol (17.22%) and γ-terpinene (12.45%) were the main compounds, and in the fruiting phase thymol (20.73%) was again the main compound, followed by γ-terpinene 14.91% and carvacrol 12.8% [26].

The differences in essential oil composition found by various authors may be explained, at least partially, by different ecological conditions and/or harvesting time of the investigated plant materials. According to literature data and our results, it is obvious that there are some differences in the essential oil composition of *S. thymbra*. However, its principal constituents were determined to be mainly carvacrol, thymol, *p-*cymene, and γ-terpinene, and very rarely α-pinene and sabinene. It has also been reported that the quantitative composition of the oil is related to the plant habitat [22]. 

**Table 1:** Chemical composition of essential oil of *S. thymbra*.

Constituents		^a^ KIE	^b^ KIL	%
	α-thujene	917.7	924	1.55 ± 0.02
	α-pinene	923.4	932	1.48 ± 0.03
	camphene	937.4	946	0.69 ± 0.01
	sabinene	963.6	969	0.14 ± 0.00
	β-pinene	965.7	974	0.70 ± 0.01
	myrcene	983.2	988	1.76 ± 0.01
	α-phellandrene	995.4	1002	0.28 ± 0.00
	δ^3^-carene	1001.2	1008	0.10 ± 0.01
	α-terpinene	**1007.8**	**1014**	**3.26** ** ±** ** 0.02**
	*p*-cymene	**1016.1**	**1020**	**7.17** ** ±** ** 0.05**
	sylvestrene	1019.5	1025	0.78 ± 0.01
	*cis*-β-ocimene	1030.6	1032	0.52 ± 0.00
	*trans-*β-ocimene	1040.6	1044	0.81 ± 0.01
	γ-terpinene	**1051.5**	**1054**	**39.23** ** ±** ** 0.27**
	*cis*-sabinene hydrate	1060.6	1065	0.23 ± 0.01
	terpinolene	1079.1	1086	0.11 ± 0.00
	linalool	1051.5	1095.6	0.34 ± 0.00
	borneol	1157.2	1165	0.82 ± 0.00
	terpinen-4-ol	1169.4	1174	0.36 ± 0.01
	α-terpineol	1185.3	1186	0.05 ± 0.01
	*neo*-dihydro carveol	n/a	1193	0.06 ± 0.04
	carvacrol methyl ether	**1235.6**	**1241**	**3.33** ** ±** ** 1.88**
	thymol	**1291.0**	**1289**	**25.16** ** ±** ** 1.95**
	carvacrol	**1297.9**	**1298**	**4.18** ** ±** ** 0.23**
	thymyl acetate	1346.9	1349	0.21 ± 0.21
	β-caryophyllene	**1407.1**	**1417**	**2.76** ** ±** ** 0.03**
	aromadendrene	1426.5	1439	0.36 ± 0.01
	α-humulene	1441.3	1452	0.15 ± 0.00
	alloaromadendrene	1448.4	1458	0.22 ± 0.22
	*cis*-β-guaiene	1484.6	1492	1.56 ± 0.02
	spathulenol	1565.9	1577	0.95 ± 0.03
	caryophyllene oxide	1570.0	1582	0.32 ± 0.01
	*cis*-α-bergamotene	^c^ n/a		0.17 ± 0.16
Total				99.81%
Yield (v/w) %				4.9
Number of constituents				33
Monoterpene hydrocarbons				58.57%
Oxygenated monoterpenes				31.21%
Sesquiterpene hydrocarbons				5.23%
Oxygenated hydrocarbons				1.27%
Other				3.53%

^a^ KIE = Kovats (retention) index experimentally determined (AMDIS); ^b^ KIL = Kovats (retention) index-literature data; ^c^ n/a-not available.

**Figure 1:**
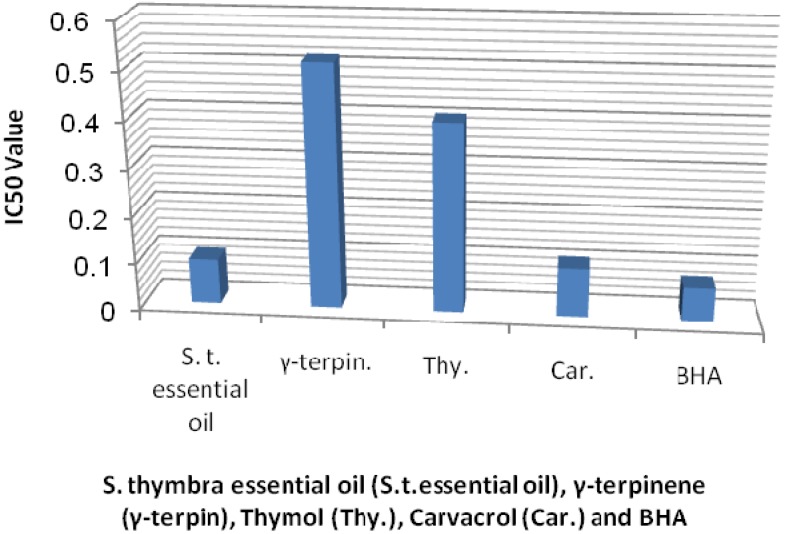
Comparisonbetween the IC_50_ values (mg/mL) of *S. thymbra* essential oil, thymol, carvacrol, γ-terpinene and BHA on DPPH assay.

Antioxidant activity was analyzed using the DPPH free radical scavenging method. The results of the DPPH scavenging assay of *S. thymbra* essential oil and its principal components and the synthetic antioxidant butylated hydroxyanisole (BHA) are shown in Figure 1. The results showed that *S. thymbra* essential oil possessed strong antioxidant activity, as seen from the concentrations at which 50% radical scavenging occurred (IC_50_ = 0.0967 mg/mL), being better than γ-terpinene and thymol and quite similar to BHA. It has been shown that the IC_50_ of *Origanum vulgare* essential oil, which was dominated by γ-terpinene and thymol (34.4% and 31.8%, respectively) was better than BHA [32]. Moreover, *O. vulgare* essential oil, containing 49% of γ-terpinene and 15% of thymol, showed an IC_50_ value quite similar to our results. Also, carvacrol, present in relative high amounts in *Thymbra capitata* oil can be partly responsible for high antioxidant activity, whereas in *O. vulgare* oil the activity may be attributed to two components, γ-terpinene and thymol [33]. According to earlier reports, some structural features, such as the presence of strongly activated methylene groups in the molecule, are probably the reason for antioxidant activity of monoterpene hydrocarbons [34].

Antibacterial activity results of the tested essential oil are presented in Table 2. The oil was active against all the bacteria tested. *S. thymbra* essential oil showed bacteriostatic effects at 0.001–0.1 mg/mL and bactericidal ones at 0.002–0.2 mg/mL. Thymol exhibited high antibacterial activity with a minimum inhibitory concentration (MIC) at 0.01–0.1 mg/mL and minimum bactericidal concentration (MBC) at 0.05–0.15 mg/mL, while carvacrol showed stronger bacteriostatic activity at 0.0025–0.05 mg/mL and bactericidal activity at 0.005–0.1 mg/mL. Inhibitory and bactericidal concentrations for γ-terpinene were slightly higher than for previous compounds (MIC at 0.05–0.2 mg/mL, and MBC at 0.07–0.3 mg/mL). Streptomycin expressed inhibitory effects at 0.0005–0.001 mg/mL and bactericidal activity at 0.0005–0.002 mg/mL. It can be seen that the essential oil showed the highest bactericidal activity (MBC = 0.002 mg/mL) against *Micrococcus flavus* and the lowest (MBC = 0.2 mg/mL) against *Listeria monocytogenes*. However, *S. thymbra* essential oil showed lower antibacterial activity than streptomycin, and was quite similar to thymol (MIC = 0.01–0.1 mg/mL and MBC= 0.05–0.15 mg/mL) and slightly lower or similar to carvacrol (MIC = 0.0025–0.025 mg/mL and MBC = 0.005–0.1 mg/mL), and it was stronger or similar to γ-terpinene (MIC at 0.05–0.2 mg/mL, and MBC at 0.07–0.3 mg/mL).

**Table 2:** Antibacterial activity of *S. thymbra* essential oil and their main compounds tested by microdilution method (MIC and MBC in mg/mL).

Bacteria	*S. thymbra*		γ-terpenene		thymol		carvacrol		streptomycin	
	MIC	MBC	MIC	MBC	MIC	MBC	MIC	MBC	MIC	MBC
Gram (+) bacteria										
*Bacillus cereus*	0.05	0.1	0.05	0.07	0.025	0.05	0.0125	0.025	0.0005	0.0005
*Micrococcus flavus*	0.001	0.002	0.05	0.07	0.025	0.05	0.0025	0.005	0.0005	0.001
*Staphylococcus aureus*	0.05	0.1	0.05	0.1	0.025	0.05	0.025	0.05	0.001	0.001
*Listeria monocytogenes*	0.1	0.2	0.1	0.2	0.1	0.1	0.05	0.05	0.001	0.002
Gram (−) bacteria										
*Escherichia coli*	0.05	0.1	0.15	0.2	0.1	0.15	0.05	0.05	0.0005	0.001
*Pseudomonas aeruginosa*	0.05	0.1	0.15	0.3	0.1	0.15	0.05	0.1	0.001	0.002
*Proteus mirabilis*	0.05	0.1	0.2	0.3	0.01	0.15	0.05	0.1	0.001	0.002
*Salmonella typhimurium*	0.05	0.1	0.1	0.2	0.05	0.1	0.05	0.05	0.001	0.001

Results of antifungal activity of compounds tested are presented in Table 3. As in the case of the antibacterial activity, the oils showed strong antifungal potential. *S. thymbra* oil possessed strong activity with (MIC) 0.001–0.025 mg/mL and showed fungicidal effects at 0.001–0.1 mg/mL. MIC and the MFC for thymol are 0.01–0.05 mg/mL, while carvacrol showed MIC at 0.0025–0.025 mg/mL and MFC at 0.005–0.05 mg/mL. MIC and (MFC) for γ-terpinene were at 0.015–0.05 mg/mL and 0.02–0.1 mg/mL. Bifonazole showed lower antifungal activity than the tested oils. MIC was at 1.5–2 mg/mL and MFC 2–2.5 mg/mL. *Penicillium ochrochloron* was the most sensitive fungus, with MBC at 0.001 mg/mL, while *Candida albicans* was the most resistant species when treated with this oil. Fungi were in general more sensitive than bacterial species (Table 2 and Table 3). In general, *S. thymbra* essential oil showed antifungal activity similar or lower than thymol (MIC and MFC 0.01–0.05 mg/mL) and lower than carvacrol (MIC at 0.0025–0.025 and MFC at 0.005–0.05 mg/mL), and it was slightly better or similar to γ-terpinene (0.015–0.05 mg/mL and 0.02–0.1 mg/mL).

**Table 3:** Antifungal activity of *S. thymbra* essential oil and their main compounds tested by microdilution method (MIC and MFC in mg/mL).

Fungi	*S. thymbra*		γ-terpenene		thymol		carvacrol		Bifonazole	
	MIC	MFC	MIC	MFC	MIC	MFC	MIC	MFC	MIC	MFC
*Penicillium* *funiculosum*	0.025	0.05	0.025	0.07	0.0125	0.025	0.0125	0.0125	2.0	2.5
*Penicillium* *ochrochloron*	0.001	0.001	0.025	0.07	0.025	0.025	0.0025	0.005	1.5	2.0
*Aspergillus fumigatus*	0.025	0.05	0.05	0.07	0.025	0.05	0.025	0.025	1.5	2.0
*Aspergillus* *niger*	0.025	0.05	0.02	0.03	0.01	0.02	0.025	0.025	1.5	2.0
*Aspergillus* *flavus*	0.025	0.05	0.02	0.03	0.01	0.01	0.005	0.01	1.5	2.0
*Aspergillus* *ochraceus*	0.025	0.05	0.015	0.02	0.01	0.015	0.005	0.01	1.5	2.0
*Candida* *albicans*	0.025	0.1	0.05	0.1	0.05	0.05	0.025	0.05	1.5	2.0
*Trichoderma viride*	0.025	0.05	0.05	0.07	0.01	0.01	0.005	0.01	2.0	2.5

*Satureja* oils represent an inexpensive source of natural antibacterial substances that exhibit potential for use in food systems to prevent the growth of foodborne bacteria and to extend the shelf life of processed foods [26]. More recently, [11] concluded that the essential oil of *S. thymbra* (1%), as well as its hydrosol fraction (100%), present sufficient bactericidal effect on bacterial biofilms formed on stainless steel suggesting that the use of natural antimicrobial agents could provide alternative or supplementary ways for the disinfection of microbial-contaminated industrial surfaces.

Previous investigations showed that the essential oil of *S. thymbra* was found to be active against the bacteria *E. coli*, *P. aeruginosa*, *S. typhimurium*, *S. sonnei* and *S. aureus* and the yeast *C. albicans* [7]. *S. thymbra* essential oil from Greece possessed very good antifungal properties with low MIC (0.1–1.0 μL/mL) and MFC (0.2–2.0 μL/mL) values [23]. The antifungal activity results of *S. thymbra* oil against *M. perniciosa,* a contaminator of *Agaricus bisporus,* obtained by the micro atmosphere method, showed MIC of 0.001–0.05 μL/mL and MFC of 0.1–0.25 μL/mL [35]. More recently, it has been shown that the oil of *S. thymbra* exhibited minimum inhibitory concentration (MIC) in the range of 0.6–5.0 μg/mL, and minimum bactericidal concentration (MBC) in the range of 2.5–10.0 μg/mL, while fungi were more sensitive with MIC and MFC at 1.25–2.5 μg/mL, and fungicidal activity in range of 2.5–5.0 μg/mL [36]. 

It is evident that there is a relationship between the high activity of tested oil and the presence of phenol components such as thymol and carvacrol. It seems likely that thymol and carvacrol interfere with the activity of cell wall enzymes like chitin synthase/chitinase as well as α- and β-glucanases of fungi [37,38]. According to literature data, the mode of action of essential oils against bacteria cannot be attributed to one specific mechanism, and not all of these mechanisms are separate targets, some are affected as a result of another mechanism being targeted [39,40]. One of important action of essential oils against bacteria is their hydrophobicity, which enables them to partition in the lipids of the bacterial cell membrane and mitochondria, disturbing the structures and rendering them more permeable and leading to leakage of cell content [41,42,43]. Although a certain amount of leakage from bacterial cells may be tolerated without loss of viability, extensive loss of cell contents or the exit of critical molecules and ions will lead to death [44]. Consequently, the high content of phenolic components may account for the high antimicrobial activity of carvacrol-type oils [45]. It should be noted that the antimicrobial activity of *S. thymbra* against the pathogenic bacteria and fungi was lower (or equal) as compared to the essential oils compounds tested here—thymol and carvacrol. Presumably, this activity is not derived only from the presence of these phenols, but part of the activity resulted from the effect of minor active constituents. Thus, synergistic or antagonistic activity between some components may affect the observed antimicrobial activity of the oils. In the study of Cosentino *et al.* [46], the high *p*-cymene content observed in Sardinian *T. capitatus* and *T. herba-barona* ‘sample a’ may have antagonized the antimicrobial action of phenols, resulting in a weaker activity of these oils, in comparation to thymol and carvacrol. This may explain in part the higher activity of thymol and carvacrol when evaluated separately. Also, some authors discussed synergistic and antagonistic effect of components and essential oils [47]. They concluded that the effects of thymol and carvacrol are responsible for the different antibacterial activities of the essential oil of various oil chemotypes. On the other hand, there are also some reports concerning a disadvantageous antagonistic effect, weakening the essential oil action as compared with its constituents. 

The oil and components tested in here showed higher antibacterial activity against Gram (+) than Gram (−) bacteria. Gram negative bacteria are, in general, more resistant than Gram positive ones [48,49,50,51,52,53,54,55]. Overall, the oils and single compounds were more active *vs.* yeasts and Gram positive bacteria than Gram negatives, has reported in similar studies [43,46,56,57,58]. 

## 3. Experimental

### 3.1. Plant Material

The samples from wild growing samples of the investigated plants were collected during the flowering stage from Bayda town in the Green Mountain region of Eastern Libya in March 2010. The plants were identified by Dr. A. Felaly, Faculty of Science, Al-Gabel Al-Garbi University Libya, and later confirmed by one of the authors (P.D.M.). The samples were dried in shadow at room temperature for 10 days. Voucher specimens where deposited in Herbarium of Institute of Botany and Botanical Garden “Jevremovac” (BEOU; voucher No. 16618).

### 3.2. Isolation of the Essential Oil

Air-dried aerial parts of *Satureja thymbra* with the wooden parts removed (100 g) were submitted to hydrodistillation, using a Clevenger-type apparatus for 3 h, according to the British Pharmacopoeia specifications [59]. The obtained essential oil was dried over anhydrous sodium sulphate, and stored in sealed dark vials kept at 4 °C. The oil yields (v/w) on a dry weight basis are reported in Table 1.

### 3.3. Gas Chromatography and Gas Chromatography-Mass Spectrometry Analysis

Qualitative and quantitative analyses of the oils were performed using GC and GC-MS. The GC analysis of the oil was carried out on a GC HP-5890 II apparatus, equipped with a split-splitless injector, attached to a HP-5 column (25 m × 0.32 mm, 0.52 µm film thickness) and equipped with a FID detector. Carrier gas flow rate (H_2_) was 1 mL/min, split ratio 1:30, injector temperature was 250 °C, detector temperature 300 °C, while column temperature was linearly programmed from 40–240 °C (at rate of 4 °/min). The same analytical conditions were employed for GC-MS analysis, where a HP G 1800C Series II GCD system equipped with a HP-5MS column (30 m × 0.25 mm, 0.25 µm film thickness) was used. Transfer line was heated at 260 °C. Mass spectra were acquired in EI mode (70 eV), in the 40–400 *m/z* range. Identification of the individual oil components was accomplished by comparison of retention times with standard substances and by matching mass spectral data with those held in the Wiley 275 Library of Mass Spectra. Confirmation was performed using the AMDIS software and literature data [60]. For the purposes of quantitative analysis area percent obtained from the FID were used as a base.

### 3.4. Antioxidant Activity (DPPH Assay)

Radical scavenging of the DPPH radical is considered to be one of the main mechanisms by which antioxidant acts in food systems. The DPPH method we used in this study was as described earlier [61]. The stable 2,2-diphenyl-1-picrylhydrazyl radical (DPPH) was used for determination of free radical-scavenging activity of the oils and their main compounds (thymol, carvacrol and γ-terpinene). A solution (0.04 mg/mL) was prepared, and then 1,800 μL of this solution was added to 200 μL of essential oils in methanol at different concentrations. The absorbance of the remaining DPPH radical was measured spectrophotometrically at 517 nm using a Jenway 6305 UV/Vis spectrophotometer (Keison Products, Chelmsford, Essex, England) after 30 min at room temperature for all samples. Methanol was used as a blank, while methanol with DPPH solution was used as a control. All determinations were taken in triplicate and special care was taken to minimize the loss of free radical activity of the DPPH. Butylated hydroxyanisole (BHA) was used as a positive control. DPPH scavenging capacity expressed in percentage (%) was calculated using the following equation: 

% inhibition = [(*A_0_* − *A_1_*) / *A_0_*] × 100

where *A_0_* is the absorbance of control sample (without essential oils), and *A_1_* is the absorbance of the samples with essential oils at different concentrations. Oil concentrations (mg/mL) providing 50% inhibition (IC_50_) was calculated from a graph plotting scavenging activity against oil concentration.

### 3.5. Antifungal Activity

For the antifungal bioassay eight fungi were used: *Aspergillus flavus* (ATCC 9643), *Aspergillus fumigatus* (human isolate), *Aspergillus niger* (ATCC 6275), *Aspergillus ochraceus* (ATCC 12066), *Penicillium funiculosum* (ATCC 36839), *Penicillium ochrochloron* (ATCC 9112), *Trichoderma viride* (IAM 5061) and *Candida albicans* (human isolate). The organisms were obtained from the Mycological Laboratory, Department of Plant Physiology, Institute for Biological Research “Siniša Stanković”, Belgrade, Serbia. The micromycetes were maintained on malt agar and the cultures stored at 4 °C and sub-cultured once a month [62]. In order to investigate the antifungal activity of the extracts, a modified microdilution technique was used [63,64,65]. The fungal spores were washed from the surface of agar plates with sterile 0.85% saline containing 0.1% Tween 80 (v/v). The spore suspension was adjusted with sterile saline to a concentration of approximately 1.0 × 10^5^ in a final volume of 100 μL per well. The inocula were stored at 4 °C for further use. Dilutions of the inocula were cultured on solid malt agar to verify the absence of contamination and to check the validity of the inoculum. Minimum inhibitory concentration (MIC) determinations were performed by a serial dilution technique using 96-well microtiter plates. The compounds investigated were added in broth Malt medium with inoculum. The microplates were incubated for 72 h at 28 °C. The lowest concentrations without visible growth (at the binocular microscope) were defined as MICs. The fungicidal concentrations (MFCs) were determined by serial subcultivation of a 2 μL aliquot into microtiter plates containing 100 μL of broth per well and further incubation for 72 h at 28 °C. The lowest concentration with no visible growth was defined as MFC indicating 99.5% killing of the original inoculum. DMSO was used as a negative control, and the commercial fungicide bifonazole was used as positive control (1–3,000 μg/mL).

### 3.6. Antibacterial Activity

The following Gram-negative bacteria were used: *Escherichia coli* (ATCC 35210), *Pseudomonas aeruginosa* (ATCC 27853), *Salmonella typhimurium* (ATCC 13311), *Proteus mirabilis* (human isolate) and the following Gram-positive bacteria: *Listeria monocytogenes* (NCTC 7973), *Bacillus cereus* (clinical isolate), *Micrococcus flavus* (ATCC 10240), and *Staphylococcus aureus* (ATCC 6538). The organisms were obtained from the Mycological Laboratory, Department of Plant Physiology, Institute for Biological Research “Siniša Stanković”, Belgrade, Serbia. The antibacterial assay was carried out by microdilution method [63,64,65] in order to determine the antibacterial activity of compounds tested against the human pathogenic bacteria. The bacterial suspensions were adjusted with sterile saline to a concentration of 1.0 × 10^5^ CFU/mL. The inocula were prepared daily and stored at +4 °C until use. Dilutions of the inocula were cultured on solid medium to verify the absence of contamination and to check the validity of the inoculum.

The minimum inhibitory and bactericidal concentrations (MICs and MBCs) were determined using 96-well microtitre plates. The bacterial suspension was adjusted with sterile saline to a concentration of 1.0 × 10^5^ cfu/mL. Compounds to be investigated were dissolved in broth LB broth (100 μL) with bacterial inoculum (1.0 × 10^4^ cfu per well) to achieve the wanted concentrations (1 mg/mL). The microplates were incubated for 24 h at 37 °C. The lowest concentrations without visible growth (at the binocular microscope) were defined as concentrations that completely inhibited bacterial growth (MICs). The MBCs were determined by serial sub-cultivation of 2 μL into microtitre plates containing 100 μL of broth per well and further incubation for 72 h. The lowest concentration with no visible growth was defined as the MBC, indicating 99.5% killing of the original inoculum. The optical density of each well was measured at a wavelength of 655 nm by Microplate Manager 4.0 (Bio-Rad Laboratories) and compared with a blank and a positive control. Streptomycin was used as a positive control (1 mg/mL DMSO). Two replicates were done for each compound and experiments were repeated three times.

## 4. Conclusions

Our results showed that *S. thymbra* essential oil exhibited strong antioxidant activity, even higher than γ-terpinene and thymol, compounds which are well known as potent antioxidants, and quite similar to commercial BHA. It can also be concluded that the essential oils of all *Satureja* species possess strong antimicrobial activities to different extents against organisms of importance to food spoilage and/or poisoning (*Salmonella*, *Listeria*, *Penicillium* and *Aspergillus*), as well as to those of interest to the medical field such as *Staphyloccocus, Apergillus fumigatus* and *Candida*. The majority of the essential oils are classified as Generally Recognized As Safe (GRAS) [66], Their use in foods as preservatives could be limited due to flavor considerations, since effective antimicrobial doses may exceed organoleptically acceptable levels. Still, there are strong consumer trends towards natural alternatives to chemical antimicrobial agents and this is supported by changes in legislation. Therefore, there is an increasing demand for accurate knowledge of the minimum inhibitory (effective) concentrations (MIC) of essential oils to enable a balance between the sensory acceptability and antimicrobial efficacy in the food matrix, preparation and possible toxicology. Addition of oils is therefore not problematic especially not when used in such a small amount as we define according to the MIC’s and MBC/MFC’s obtained in this study. In view of broad activity, this essential oil may find industrial applications as natural preservatives and conservation agent in the cosmetic and/or food industries and as active ingredient in medical preparations.
